# CA 15–3 cell lines and tissue expression in canine mammary cancer and the correlation between serum levels and tumour histological grade

**DOI:** 10.1186/1746-6148-8-86

**Published:** 2012-06-22

**Authors:** Elisabetta Manuali, Antonio De Giuseppe, Francesco Feliziani, Katia Forti, Cristina Casciari, Maria Chiara Marchesi, Eugenio Pacifico, Karol M Pawłowski, Kinga Majchrzak, Magdalena Król

**Affiliations:** 1 Laboratory of Histopathology and Electron Microscopy, Istituto Zooprofilattico Sperimentale dell’Umbria e delle Marche, via G. Salvemini, 1 – 06126, Perugia, Italy; 2 Department of Veterinary Pathology, Diagnostic and Clinic, Faculty of Veterinary Medicine, University of Perugia, via S. Costanzo, 4 – 06126, Perugia, Italy; 3 Hematology and Clinical Pathology Service, S. Maria della Misericordia - Perugia Hospital, S. Andrea delle Fratte, Perugia, 06156, Italy; 4 Department of Physiological Sciences, Faculty of Veterinary Medicine, Warsaw University of Life Sciences - WULS, Nowoursynowska 159, Warsaw, 02-776, Poland

## Abstract

**Background:**

Mammary tumours are the most common malignancy diagnosed in female dogs and a significant cause of mortality and morbidity in this species. Carbohydrate antigen (CA) 15–3 is a mucinous glycoprotein aberrantly over-expressed in human mammary neoplasms and one of the most widely used serum tumour markers in women with breast cancer. The aim of this study was to investigate the antigenic analogies of human and canine CA 15–3 and to assess its expression in canine mammary cancer tissues and cell lines. Immunohistochemical expression of CA 15–3 was evaluated in 7 canine mammary cancer cell lines and 50 malignant mammary tumours. As a positive control, the human breast carcinoma cell line MCF7 and tissue were used. To assess CA 15–3 staining, a semi-quantitative method was applied. To confirm the specificity and cross-reactivity of an anti-human CA 15–3 antibody to canine tissues, an immunoblot analysis was performed. We also investigated serum CA 15–3 activity to establish whether its expression could be assigned to several tumour characteristics to evaluate its potential use as a serum tumour marker in the canine mammary oncology field.

**Results:**

Immunocytochemical analysis revealed CA 15–3 expression in all examined canine mammary cancer cell lines, whereas its expression was confirmed by immunoblot only in the most invasive cells (CMT-W1, CMT-W1M, CMT-W2 and CMT-W2M). In the tissue, an immunohistochemical staining pattern was observed in 34 (68%) of the malignant tumours. A high statistical correlation (p = 0.0019) between serum CA 15–3 levels and the degree of tumour proliferation and differentiation was shown, which indicates that the values of this serum marker increase as the tumour stage progresses.

**Conclusions:**

The results of this study reveal that CA 15–3 is expressed in both canine mammary tumour cell lines and tissues and that serum levels significantly correlate with the histological grade of the malignancy.

## Background

Spontaneous mammary tumours are commonly observed in female dogs, particularly in many European countries where animals are not routinely spayed at a young age. The majority of malignant tumours arise from epithelial tissue and metastasise to the lungs or other organs [1].

Several histopathological variables, such as tumour size, lymph node status, lymphatic or vascular invasion and the tumour grade of differentiation form, have been widely used parameters in prognostic evaluations [2,3].

In human breast cancer and other malignancies, serum tumour markers play an important role in patient management [4,5]. Tumour cells display protein molecules on their surfaces. These proteins, called tumour-associated antigens, may be present in higher than usual concentrations in other tissues, serum, urine or body fluids of patients with cancer. Thus, the elevation of these markers may be helpful in early diagnosis, determining prognosis, following a course of treatment, predicting the response or resistance to specific therapies and surveillance after primary surgery [6,7].

Carbohydrate antigen (CA) 15–3 (Mucin 1, MUC1), the product of the *mucin 1* gene, is a large transmembrane glycosylated molecule aberrantly overexpressed in many adenocarcinomas in an underglycosylated form and then shed into the circulation [8,9]. The antigen bears epitopes that are recognised by two murine monoclonal antibodies: the high molecular weight mucin-like glycoprotein DF3 (*mucin 1* gene-derived), raised against a membrane fraction of liver metastasis from breast cancer, and 115d8, raised against a milk fat globule membrane [10,11]. During a malignant transformation, the membrane expression of the MUC1 cell surface-associated oncoprotein often changes from apical to circumferential simultaneously with a loss of polarity of the epithelial cells, acting as anti-adhesive molecules and facilitating the detachment of malignant cells, and increasing the metastatic and invasive potential of tumour cells [12]. Currently, CA 15–3, the circulating form of DF3 antigen, is one of the most widely used serum biochemical markers in breast cancer. High serum levels in pre-operative patients correlate with large-size tumours, the stage of the disease and the presence of lymph node metastases and are associated with an adverse patient outcome [13,14].

In spite of the advanced diagnostic tools available in human oncology, the methodologies available in veterinary medicine may still be considered to be in progress, and there is a need to develop reliable and easily performed diagnostic tests that may predict an increasing tumour burden or recurrence after a mastectomy in the individual patient and prove more useful in terms of classification and prognosis.

Currently, there is a lack of scientific documentation regarding the application of serum tumour markers in the canine mammary oncology field, and only one preliminary report describing the possible use of serum CA 15–3 in dogs has been published [15]. The Authors showed that the application of direct chemiluminescence using a human commercial kit appears to be a promising method for the determination of CA 15–3 levels in canine mammary oncology.

The present study was therefore designed to verify the antigenic correlation of human and canine CA 15–3, to assess the expression profile of this tumour-associated antigen and to establish whether its expression could be associated with several tumour characteristics to evaluate its potential use as a serum tumour marker in the canine mammary oncology field.

## Methods

### Cell lines

#### Cell cultures

The seven canine cell lines used for this study have previously been recounted [16-19]. Two of the cell lines used in this study originated from mammary adenocarcinomas (CMT-W1 and CMT-W2), and two of the cell lines originated from their metastases to the lungs (CMT-W1M and CMT-W2M, respectively); these cell lines were kindly donated by Prof. Dr. Maciej Ugorski and Dr. Joanna Polańska of the Wrocław University of Environmental and Life Sciences in Poland. One cell line originated from canine mammary anaplastic cancer (P114) and was kindly provided by Dr. Gerard Rutteman of Utrecht University in the Netherlands. Two canine cancer cell lines were kindly provided by Dr. Eva Hellmen of the Swedish University of Agricultural Sciences (Sweden): mammary carcinoma (CMT-U27) and spindle-cell mammary tumour (CMT-U309). The cell lines were routinely cultured in RPMI-1640 medium (Sigma Aldrich, USA) enriched with 10% foetal bovine serum (FBS) (Gibco, USA), penicillin-streptomycin (50 IU/ml) and fungizone (2.5 mg/ml) (Sigma Aldrich, USA) under conditions of 5% CO_2_/95% humidified air at 37 °C.

Human breast adenocarcinoma cell line (MCF7) was kindly provided by Dr. Luisa Lanfrancone of Campus IFOM-IEO Milano (Italy). This cell line was grown in Dulbecco’s modified Eagle’s medium (DMEM) (Gibco, USA) supplemented with 10% FBS (Gibco, USA), 100 IU/ml penicillin and 100 μg/ml streptomycin (Sigma Aldrich, USA) at 37 °C in a humidified atmosphere with 5% CO2.

#### Immunocytochemistry (ICC)

Cells were cultured on Lab Tek (Nunc Inc., DK) 4-chamber culture slides and were fixed with 70% ethanol after 24 hours. After washing with Tris buffered saline (TBS) (Dako, DK), the samples were incubated in Peroxidase Blocking Reagent (Dako, DK) for 10 min at room temperature. After a 30 min incubation in TBS with 5% bovine serum albumin (BSA) (Sigma Aldrich, USA), the anti DF3 (Imgenex, USA) and anti 115d8 (Abcam, UK) monoclonal antibodies, diluted 1:100 in 1% BSA, were used, and then the slides were incubated at +4 °C overnight. For staining, the EnVision kit (Dako, DK) was used. To develop the coloured product, the 3,3`-Diaminobenzidine (DAB) substrate (Dako, DK) was used. Finally, haematoxylin was used for nuclei counterstaining.

For immunocytochemical experiments, the negative control samples stained without the use of primary antibodies were set aside. Four independent experiments were conducted.

Ten pictures of each slide were taken using Olympus microscopy BX60 (Olympus, DE). The colorimetric intensity of the ICC-stained antigen spots (brown colour) was measured by a computer-assisted image analyser (Olympus Microimage™ Image Analysis, software version 4.0 for Windows, USA). The antigen spot colour intensity is expressed as the mean pixel optical density on a 1–256 scale.

#### Invasion assay and 3D culture

The invasion assay and 3D culture of the canine mammary cancer cell lines CMT-U27, CMT-U309, P114, CMT-W1 and CMT-W2 has been previously reported [20]. BD BioCoat Matrigel™ invasion chambers (BD Biosciences, USA) pre-coated with BD Matrigel matrix were used according to the manufacturer’s protocol. The assay insert plates were prepared by rehydrating the BD Matrigel Matrix coating with phosphate buffered saline for two hours at 37 °C. The rehydration solution was carefully removed, and 2.5x10^5^ cancer cells were added to each apical chamber and 0.75 ml of RPMI-1640 containing chemo attractant (10% FBS) was added to the basal chamber. Uncoated insert plates, included as invasion controls, were used without rehydration. Assay plates were incubated for 22 hours at standard culturing conditions. Then, 2.5 μg/ml calcein AM was added to 20 μl DMSO, and 10 μl of this solution was transferred to 12 ml Hanks Buffered Saline Dispense. Next, 0.5 ml calcein solution was transferred into each well of a 24-well plate. The medium from the insert was removed, and multiwell inserts were transferred to the plate containing 0.5 ml/well calcein. Plates were incubated for an hour at standard culture conditions. The fluorescence of the invaded cells was measured with an excitation wavelength of 485 nm and an emission wavelength of 530 nm using a Tecan Infinite 200 Reader (Tecan, USA).

To characterise the growth of the cancer cell lines, they were treated with trypsin and resuspended in the culture medium. Thirty-five-mm culture plates (Corning Inc.) were coated with 100 μl of growth factor reduced Matrigel (BD Biosciences, USA) and left to solidify for 30 min at 37 °C. The cells were then plated at a concentration of 10^4^ cells/ml. The growth of cells on Matrigel was observed each day under a phase-contrast microscope.

#### SDS-PAGE and western blot

The cells were pelleted by centrifugation at 400 *x* g at 4 °C for 5 min. Protein extracts from cultured cells were isolated by lysis of the collected pellets with RIPA buffer (50 mM Tris, pH 7.5, 150 mM NaCl, 1 mM EDTA, 1% NP-40, 0.25% Na-deoxycholate and 1 mM PMSF) supplemented with a protease inhibitor cocktail (Sigma-Aldrich, USA) and a phosphatase inhibitor cocktail (Sigma-Aldrich, USA) for 30 min at 4 °C. Lysates were cleared for 20 min at 16,000 *x* g, and supernatants were collected. The protein concentration in the lysates was determined by Bio-Rad Protein Assay Dye (Bio-Rad Laboratories Inc., USA). Cellular proteins extracted from canine and human mammary cancer cell lines were analysed by SDS-PAGE and subjected to immunoblotting analysis. Samples were mixed with 4x NuPage sample buffer (Invitrogen, CA) containing 100 mM DDT and heated to 95 °C for 5 min. Lysates were subjected to electrophoresis using 3-8% NuPage Tris-Acetate precasting gels in Tris-Acetate SDS Running buffer and transferred to a PVDF membrane according to the manufacturer’s protocol. Membranes were blocked for 2 hours at room temperature in Phosphate Buffered Saline (PBS), pH 7.2, containing 0.05% Tween 20 (PBST) and 3% skimmed milk. Membranes were then washed with PBST and incubated at 4 °C overnight with the anti DF3 (Imgenex, USA) and 115d8 (Abcam, UK) monoclonal antibodies, diluted 1:100 in 1% milk/PBST and anti-ß-actin antibodies (Santa Cruz Biotechnology, USA) diluted 1:1.000 in 1% milk/PBST (as a reference protein). Detection was performed by the SuperSignal West Pico Chemiluminescence Substrate using an anti-mouse antibody conjugated with horseradish peroxidase (Pierce Biotechnology, USA).

### Animals, serum biochemistry, histology and immunohistochemistry

Fifty tissue samples were obtained from female dogs with malignant mammary tumours during surgery in private clinics; 27 were solitary nodules, and 22 were multiple nodules presenting simultaneously with 2 or more lumps. In one case, data were not available.

To select only primary tumours without lymph node involvement and lung metastasis, the female dogs were initially submitted to a systematic clinical examination together with a macroscopic evaluation of the tumour and the lymph nodes to accomplish a staging categorisation. Several clinical factors, such as tumour size, the presence/absence of tumour metastasis to regional lymph nodes and the presence/absence of distant metastases, were used as a staging system (TNM), according to Philbert [21]. As part of the diagnostic workup, the altered regional lymph nodes were evaluated by means of a tough fine needle aspirate (FNA) and cytology. Thoracic radiological examinations searching for lung metastases were also performed. A routine clinicopathological examination (blood cell count, serum biochemical profile and urinalysis) to exclude specific benign conditions that could influence the serum levels of CA 15–3 (acute hepatitis, chronic liver diseases, chronic renal failure, and dermatological conditions) was also performed in all animals. To determine factors that possibly influence serum CA 15–3 activity in cancer patients, the impact of clinical and pathological parameters were investigated and cases were assessed for tumour size, skin ulceration, histological type, tumour grade, inflammation and necrosis. Unfortunately, the data for the length of survival time were unavailable for most patients.

Peripheral venous blood samples were obtained from the patients during the pre-operative stage to measure CA 15–3. The anticoagulated whole blood from patients subjected to a mastectomy was collected for routine diagnostic purposes. The remaining volume of the blood samples was collected for our analyses (with the written permission of the dogs’ owners). Sera were separated and stored at −20 °C until use. CA 15–3 levels were determined by a direct chemiluminescent technology in the automated immunoanalyser system ADVIA Centaur® using a kit for human diagnostic oncology (Bayer Immuno 1 CA 15-3™ assay) according to the manufacturer's protocol. Values were expressed as Units/ml (U/ml). As a control group, we used data obtained in our previous study [15].

For histological examinations, representative portions of tissue samples were fixed in 10% buffered formalin and then dehydrated and embedded in paraffin. Three to 4-micron-thick consecutive sections were cut and stained with haematoxylin and eosin (HE) and subjected to the immunohistochemical procedure using the anti MUC1 antibodies previously described. To better investigate the pattern of expression on canine mammary tissues, the immunohistochemical procedure was also applied on 5 normal lesions and on 10 benign breast lesions (3 complex adenomas, 2 simple adenomas, 2 benign mixed tumours and 3 dysplastic lesions) obtained from the archive of the Laboratory of Histopathology of the Istituto Zooprofilattico Sperimentale dell’Umbria e delle Marche.

In HE sections, tumours were classified according to the WHO’s criteria for canine mammary neoplasms [3] and coded according to the WHO’s International Classification of Disease for Oncology System (ICD-O). In cases of multiple nodules in a chain, the entire chain was resected, and all nodules were subjected to histology. Only the tumour with the higher grade of malignancy was used as the basis for a comparison with CA 15–3.

Histological grading was evaluated in accordance with Elston and Ellis [22], the most current method for the histological grading of human breast cancers, recently applied to canine mammary tumours and more practical for the comparison of data between researchers [23-25]. According to this method, histological findings were recorded and used to classify mammary carcinomas as grade I, grade II or grade III tumours.

For immunohistochemistry (IHC), tissue sections were hydrated utilising xylene and a graded alcohol series. Successively, the quenching of endogenous peroxidase activity was achieved by incubating the sections for 30 min in 3% hydrogen peroxide in methanol at room temperature. Antigen retrieval was performed using microwave exposure for 5 min at 650 W (three times) and 500 W (two times) in a citrate buffer solution (pH 6.0). Primary antibodies (diluted 1:50 in PBS) were applied and incubated overnight at 4 °C in a humidified chamber. A streptavidin-biotin revelation commercial kit (Dako, DK) was used, and the reaction was developed by 3,3’-diaminobenzidine (DAB). The slides were washed in tap water and counterstained with Mayer’s haematoxylin. A positive control of human malignant breast cancer tissue was included in each staining. Negative control slides were incubated with a specific non immune antibody in parallel with each staining batch to confirm the antibody specificity.

To evaluate MUC1 expression, a semi-quantitative method was performed; protein expression was assessed estimating the percentage of labelled cells and scored as follows: no staining, weak staining (≤20%), moderate (≥20 ≤50%) and strong (≥50%) immunostaining. The images were digitalised using a video camera connected to a microscope (DMR Fluo HC, Leica, USA), and the semi-quantitative evaluation was estimated by counting 1.000 positive cells in randomly selected fields (magnification 200x), avoiding necrosis.

### Statistical analysis

The statistical analysis was conducted using Prism version 5.00 software (GraphPad Software, USA). The ANOVA and post-hoc Dunett`s Multiple Comparison test and Tukey’s Honestly Significance Difference (HSD) were applied. To assess the correlation between the tumour characteristics and the CA 15–3 serum level, the Pearson correlation coefficient was used. A p < 0.05 was regarded as significant, whereas p < 0.001 was regarded as highly significant.

## Results

### Cell lines

#### Immunocytochemistry

Each tumour cell line used in this study showed cytoplasmic expression of MUC1, and the positivity was mainly observed in a granular pattern. Frequently, cytoplasmic staining was observed together with a membrane reaction in a mixed pattern. CMT-W1, CMT-W1M, CMT-W2 and CMT-W2M cell lines showed a high expression of MUC1, whereas CMT-U27, CMT-U309 and P114 cell lines showed weak 115d8 expression. The same results were obtained using antibodies against DF3 (data not shown).

**Figure 1:**
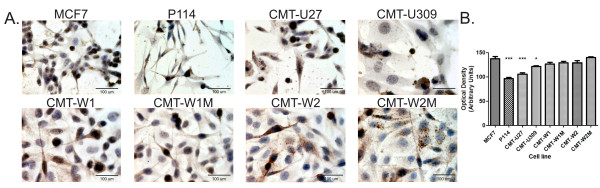
**MUC1/CA 15–3 expression in canine mammary cancer cell lines and the human MCF7 cell line.A**. Pictures of MCF7 human mammary carcinoma cells and canine mammary cancer cell lines: P114, CMT-U27, CMT-U309, CMT-W1, CMT-W1M, CMT-W2 and CMT-W2M obtained with an Olympus BX60 microscope (at the magnification of 200x). The MUC1 antigen is reflected as a brown colour. **B**. The graph of the mean MUC1 expression (and SE) in the examined cell lines. Ten pictures in each slide were analysed. The brown colour intensity was counted by a computer-assisted image analyser (Olympus Microimage™ Image Analysis, software version 4.0 for Windows, USA). The statistical analysis was performed using Prism version 5.00 software (GraphPad Software, USA). The ANOVA and Dunett`s Multiple Comparison post-hoc test were applied to analyse the MUC1 expression in the examined cell lines (the MCF7 cell line was regarded as a control); p < 0.001 was regarded as highly significant and marked as ***, whereas p < 0.05 was regarded as significant and marked as *.

The mean optical density (Arbitrary Units) of brown coloured antigen reflecting 115d8 expression was counted. As a control, the MCF7 cell line was used, which expresses the antigen at the level of 137.6 (±SE: 4.27). No significant differences were found between the expression of the DF3 antigen in the control cell line and in the following cell lines: CMT-W1 (124.7; ±SE:3.65), CMT-W1M (128.8; ±SE: 3.25), CMT-W2 (129.9; ±SE: 4.39), and CMT-W2M (140.2; ±SE: 1.47). A significant difference (p < 0.05) was found between the 115d8 expression in the MCF7 cell line and the CMT-U309 cell line (121.6; ±SE: 1.57), whereas highly significant differences (p < 0.001) were found in the 115d8 expression in the CMT-U27 and P114 cell lines (105.3; ±SE: 3.36 and 96.57; ±SE: 2.30, respectively) in comparison with MCF7 (Figure 1).

#### Invasion of canine mammary cancer cell lines

**Figure 2:**
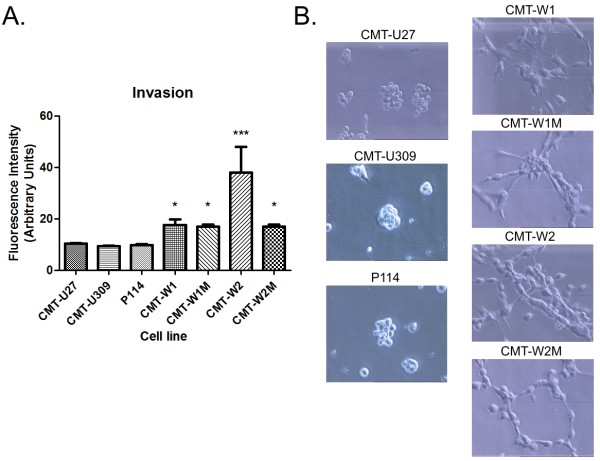
**Invasion of canine mammary cancer cell lines.A**. The graph of fluorescence intensity related to the invasion of canine mammary cancer cell lines: CMT-U27, CMT-U309, P114, CMT-W1, CMT-W1M, CMT-W2 and CMT-W2M. The statistical analysis was performed using Prism version 5.00 software (GraphPad Software, USA). The ANOVA + Tukey’s test were applied to analyse the results. p < 0.05 was regarded as significant and marked as *, whereas p < 0.001 was regarded as highly significant and marked as ***. **B**. Growth characteristics of the CMT-U27, CMT-U309, P114, CMT-W1, CMT-W1M, CMT-W2 and CMT-W2 cell lines (phase contrast micrographs) grown on Matrigel matrix for 24 hours.

The invasion assay results and growth characteristics on the Matrigel matrix of the canine mammary cancer cell lines CMT-U27, CMT-U309, P114, CMT-W1 and CMT-W2 have previously been reported [20]; however, these cell lines have not been compared with CMT-W1M and CMT-W2M cell lines. The assay showed that only CMT-W1, CMT-W1M, CMT-W2 and CMT-W2M cells migrated through the Matrigel (Figure 2A). The fluorescence intensity related to the migration of the CMT-W1 cells was 17.57 (p < 0.05 compared with non-migrating cells), and the migration of the CMT-W1M cells was 17.00 (p < 0.05 compared with non-migrating cells), whereas migration of the CMT-W2 and CMT-W2M cells was 38.00 (p < 0.001 compared with non-migrating cells) and 17.00 (p < 0.05 comparing with non-migrating cells), respectively.

To confirm the ability of these cell lines to invade the matrix, we have assessed their growth characteristics on the Matrigel matrix (Figure 2B). After 22 hours of culturing (similarly as in the invasion assay) on Matrigel, the CMT-U27, CMT-U309 and P114 cell lines formed colonies, whereas the CMT-W1, CMT-W1M, CMT-W2 and CMT-W2M cell lines formed branching structures (Figure 2B), which indicated their invasive phenotype.

#### SDS-PAGE and western blot

**Figure 3:**
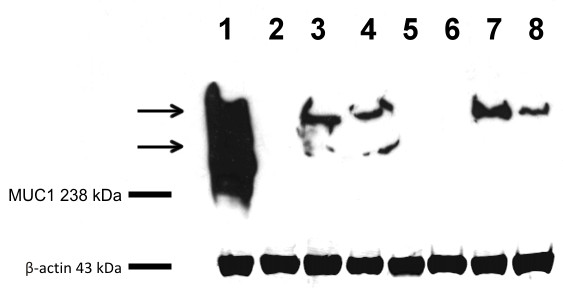
**Western blot analysis of the cellular extracts from human and canine mammary cell lines.** Representative Western blot picture of MUC1 expression in the examined cancer cell lines: Lane 1: human breast cancer cell line MCF-7; Lane 2: canine spindle-cell tumour cell line CMT-U309; Lanes 3 and 4: canine mammary adenocarcinoma cell lines CMT-W1 and CMT-W2; Lane 5: canine mammary carcinoma cell line CMT-U27; Lane 6: canine mammary anaplastic cancer cell line P114; Lanes 7 and 8: canine mammary metastatic cancer cell lines CTM-W1 and CTM-W2.

Immunoblot analysis using anti 115d8 antibodies revealed the presence of two reactive protein bands in the human MCF7 cell line, used as a positive control (indicated by arrows in Figure 3 lane 1). The band with a lower molecular weight (MW) is approximately 250 kDa, while the MW of the major protein band appears to be more than 300 kDa. A single protein band, with the same high MW as the positive control, was detected for the CMT-W1 and CMT-W2 cell lines (Figure 3, lanes 3 and 4), as well as for the CMT-W1M and CMT-W2M cell lines (Figure 3, lanes 7 and 8). No bands were observed for the CMT-U309, CMT-U27 and P114 cell lines (Figure 3, lanes 2, 5 and 6). Immunoblotting performed with the anti DF3 antibody gave overlapping results (data not shown).

The Western blot analysis with anti ß-actin antibodies showed that variability in protein loading could not account for the observed differences in 115d8 expression.

### Animals, serum biochemistry, histology and immunohistochemistry

Most dogs in our series were mixed breeds (32.3%), followed by German Shepherd dogs (11.8%). The mean age was 9.2 ±2.4 years (range 3–17), with an increased prevalence among intact females (79.4%). The mean diameter of nodules was 3.09 ± 2.90 cm (range 0.5-12 cm), and skin ulceration was present in 22% of cases. None of these patients had apparent nodal involvement.

With respect to the histopathological analyses based on the WHO’s classification, complex carcinoma was the most common type of tumour (68%) followed by simple carcinoma (28%) and carcinosarcoma (4%); necrosis and inflammation were observed in 62% and 42% of cancer cases, respectively. According to Elston and Ellis’s method, 66% of tumours were classified as grade I, 28% were classified as grade II and 6% were classified as grade III.

CA 15–3 was detected in all of the analysed sera. Furthermore, dogs with malignant mammary tumours had a significantly higher median activity of serum CA 15–3 (0.80 ± 0.55) than the control group (0.57 ± 0.21) (p = 0.004). When we compared tumour Carbohydrate antigen 15–3 serum levels and the pathological variables considered in this study, no statistically significant differences between CA 15–3 and age, history of ovariohysterectomy, numbers and sizes of tumour and histological types were observed (Table 1). Furthermore, because the inflammatory and necrotic processes were often associated with cancer, they did not appear to have influenced the serum levels of CA 15–3. Interestingly, a highly significant correlation was found between CA 15–3 serum levels and the tumour grade (p <0.0019). In grade I tumours, the average concentration of this marker was approximately half (0.64 U/ml) compared with that of grades II (1,21 U/ml) and III (1,33 U/ml). Given the fact that grade III neoplasms were represented by a small number (n. 3 cases) and that their corresponding mean serum level was not significantly different from grade II tumours, they were included in the same group.

**Table 1:** The relation of CA 15–3 serum concentration to the clinical-pathological variables analysed

		**Serum CA 15–3 levels (mean ± SD)**	**p-Value**
**Tumour size (cm)**	<2	0.70 ± 0.67	n.s.
	2-5	0.84 ± 0.45	n.s.
	>5	1.03 ± 0.54	n.s.
**Skin ulceration**	Yes	0.54 ± 0.15	n.s.
	No	0.91 ± 0.63	n.s.
**Histopathology**	Simple carcinoma	0.63 ± 0.40	n.s.
	Complex carcinoma	0.78 ± 0.57	n.s.
	Carcinosarcoma	1.50 ± 0.28	n.s.
**Inflammation**	Yes	0.64 ± 0.46	n.s.
	No	0.90 ± 0.60	n.s.
**Necrosis**	Yes	0.80 ± 0.59	n.s.
	No	0.80 ± 0.53	n.s.
**Histological grade**	Grade I	0.64 ± 0.36	n.s.
	Grade II	1.21 ± 0.79	0.0019
	Grade III	1.33 ± 0.32	0.0019

Relation between tumour size, skin ulceration, histopathological diagnosis, inflammation, necrosis and histological grade of canine mammary cancer tissues and serum CA 15–3 levels. The Pearson correlation coefficient was applied using Prism version 5.00 software (GraphPad Software, USA).

**Figure 4:**
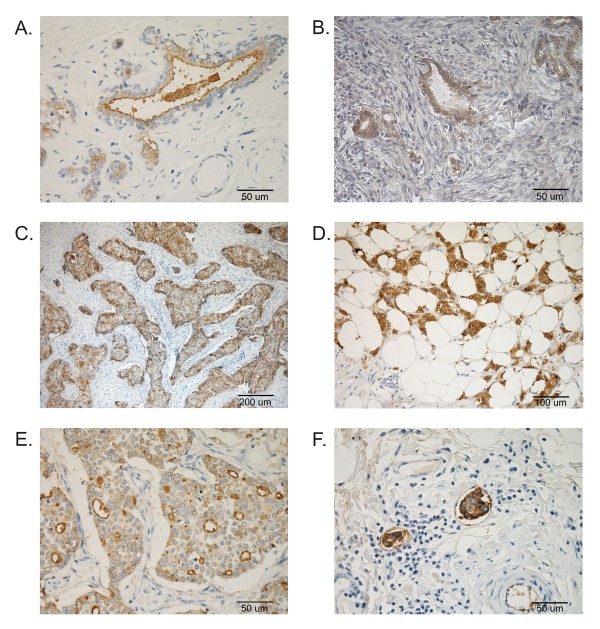
**MUC1 expression in the canine normal mammary gland and in benign and malignant mammary lesions.** Sections of specimens stained immunohistochemically. MUC1 immunoreactivity was present in normal tissues samples, showing luminal and apical expression (**A**). Moderate and heterogeneous labelling of epithelial cells in complex adenoma (**B**). Higher level of immunoreactivity in complex carcinoma (**C**), in cancer cells invading the adipose tissue (**D**), in simple carcinoma (**E**) and in intra lymphatic metastatic cells (**F**). The images were digitalised using a video camera connected to a microscope (DMR Fluo HC, Leica, USA), and the semi-quantitative evaluation was estimated by counting 1.000 positive cells in randomly selected fields (magnification 200x) avoiding necrosis.

 With immunohistochemistry, DF3 antigen was detected in all normal gland tissues, and, as expected, the immunoreactivity was almost restricted to the apical membrane of epithelial cells, which displayed a weak to moderate staining. The positivity was more evident in ductal epithelial cells and in luminal secretions (Figure 4A).

In benign mammary lesions (hyperplastic and neoplastic), the staining was observed in 7/10 (70%) of the analysed cases, and the pattern of reaction was weak-to-moderate, usually restricted to the apical membrane of the epithelial cells. Approximately 20% of normal ducts also showed a slight apical staining. In complex adenomas, simple adenoma and benign mixed tumours, a cytoplasmic stain was also detected (Figure 4B), while in the dysplastic lesions (n.1 adenosis and n. 2 fibrocystic disease), no evidence of reaction was found.

In malignant mammary tumours, the apical staining was almost always observed together with a cytoplasmic expression in a mixed pattern with variable intensity and mostly in diffuse or granular pattern. It is important to note that the reaction affected all of the cells in 34 (68%) carcinoma samples, including 20 (59%) with strong and 14 (41%) with moderate staining. In complex carcinomas, epithelial cells showed a diffuse positivity in 24 (70.6%) cases, with a greater intensity at the periphery of the neoplastic foci (Figure 4C). In the marginal areas of invasive tumours, markedly positive neoplastic cells invading the surrounding adipose tissue were frequently observed (Figure 4D). In simple carcinomas, a diffuse expression was detected in 12 (85.7%) cases and most evident in the apical membrane of neoplastic cells that formed tubular structures (Figure 4E). This tumour had a strong tendency to infiltrate the surrounding vessels, and lymphatic emboli, when present, exhibited a strong cytoplasmic positivity (Figure 4F). In carcinosarcomas, DF3 expression was very weak in 100% of cases because the sarcomatous cells were preponderant and only a few stained epithelial cells were observed. In hyperplastic or normal lobules that sometimes circumscribed the tumour proliferated areas, a weak positivity was also detected. In all of the tissues examined, myoepithelial and stromal cells, lymphocytes, macrophages and blood vessels did not express immunoreactivity. No significant correlations were found between DF3 overexpression and serum CA 15–3 levels.

## Discussion

In veterinary medicine, a variety of assays and techniques have been used in an attempt to identify the prognostic markers for neoplastic diseases, including clinical parameters, histologic features, immunohistochemistry, quantitative PCR, evaluation for genomic mutations and specific serum protein levels or enzyme activity analyses [26].

To investigate the potential functional relevance of CA 15–3 as a serum tumour marker in veterinary oncology, we performed a multidisciplinary study on canine neoplastic mammary tissues and cell lines.

The main finding in Western blot analysis regarded the positive interaction between the human antibodies and the canine MUC1 protein. The protein sequence of epitope DF3 in humans is TRPAPGS [27], whereas the canine DF3 epitope sequence is SRPAPSS (protein sequences have been obtained from NCBI with accession numbers [canine NP_001181906.1 and human P15941.3] and then compared using the MultAlin on-line platform [28]). We used the BLOSUM62 amino acid substitution matrix [29,30] to score alignments between evolutionarily divergent protein sequences. It revealed a high similarity between these two epitopes in humans and in dogs (with only one point of difference). Thus, theoretically, the similarity of the protein sequence of the DF3 epitope is very high in these two species. Nevertheless, the results of WB showed that anti 115d8 and anti DF3 antibodies display cross-reactivity with canine samples. The ICC analysis of the MUC1 expression (using both antibodies) in canine mammary cancer cell lines showed staining patterns similar to those observed in humans.

In WB, a double protein band with a high molecular weight was observed in MCF-7 cells [31-33]. In the canine tumour cell lines CMT-W1, CMT-W2, CMT-W1M and CMT-W2M, we found a single protein band of MUC1 with a molecular weight similar to the higher human form. However, when we extended the time of the chemical exposure, some films revealed an apparent reactive point with a molecular weight of approximately 250 kDa. These data are still under investigation because it is difficult to determine whether it is an actual protein band or an artefact of the analysis, considering the low sensitivity of the test.

Our immunocytochemistry results differ from those obtained using Western blot. The immunocytochemical examination revealed MUC1 expression in all of the examined cell lines. However, only the CMT-W1, CMT-W1M, CMT-W2 and CMT-W2M cell lines expressed MUC1 at high levels, similar to MCF7 (no statistically significant differences were found). The remaining cell lines exhibited a weaker expression of MUC1, which differed significantly from the MCF7 cell line. According to the subject data [34,35], a higher expression of MUC1 is observed in more invasive metastatic cancer. Our findings support this hypothesis, as CMT-W1, CMT-W1M, CMT-W2 and CMT-W2M cell lines with invasive phenotypes [17,18,20] (Figure 2) showed a higher MUC1 expression (detected in WB and ICC). Thus, a lack of MUC1 expression in non-invasive P114, CMT-U27 and CMT-U309 cell lines in Western blot analysis may be due to low levels of this antigen expression and/or the low sensitivity of the Western blot analysis. Although both methods detect a specific protein expression, they significantly differ from each other on what may explain the differences in the obtained results. Similar variations between the results obtained using Western blot and immunocytochemistry have previously been reported [36], showing that immunocytochemistry is a more sensitive method than the Western blot in cases of weak antigen expression.

Statistical analysis allowed us to demonstrate that CA 15–3 serum levels correlate positively with the degree of tumour proliferation and differentiation, as a significantly higher concentration of CA 15–3 was found in grades II-III than in grade I carcinomas (p = 0.0019). Tumour size, skin ulceration, necrosis, inflammation and the histological type of mammary cancer were not significantly related to the serum levels of CA 15–3.

The existence of a relationship between CA 15–3 serum levels and tumour grading appears to be logical because we assumed that tumour markers reflect the number and the activity of neoplastic cells as well as their products that access the circulation where the marker is measured.

Therefore, this parameter could be taken into consideration as potentially enabling a better evaluation of a tumour’s malignancy, particularly for establishing the preoperative stage of the neoplasm and providing useful guidelines when determining the surgical approach.

Another finding of our study, as expected, is the expression of MUC1 in normal canine mammary tissues and in mammary gland benign lesions. The reaction was primarily restricted to the apical membrane of epithelial cells. However, in benign tumours, the pattern of reaction was extended to the cytoplasm of the luminal epithelial cells. These results are in absolute parallel to those observed in human mammary tissues. [37].

Among the carcinomas, we observed MUC1 activity in 68% of the analysed cases that displayed a strong immunostaining in a mixed pattern (involving the entire cell surface and the cytoplasm), which comprises the whole cell population in several carcinoma samples. This finding is in accordance with the observation of Lacunza [38], which demonstrated a correlation between MUC1 protein over-expression and gene amplifications in benign and malignant breast tumours. Many studies have revealed that this event could be linked to the presence of membrane-associated MUC1 molecular portions that are shed from the surface of tumour cells to the cytosol by modulation of its glycosylation state [39,40].

In the present study, there was no significant correlation between the over-expression of MUC1 and CA 15–3, the circulating form of the DF3 antigen. However, a significant correlation has been found between the histopathological grade of the tumour malignancy and MUC1 expression and between the histopathological grade of the tumour malignancy and the circulating CA 15–3 level. We suppose that a lack of a correlation between MUC1 over-expression in tissues and the serum level of CA 15–3 may be related to the different antibodies used in the IHC and CA 15–3 tests.

## Conclusions

To the best of our knowledge, this is the first report demonstrating a significant correlation between serum CA 15–3 expression and tumour malignancy in dogs with mammary cancer. Our results may provide the basis for developing a novel approach in veterinary research, although additional studies using a large number of healthy and pathological clinical samples to validate these preliminary results and to establish normal and pathological serum ranges of CA 15–3 antigen in dogs.

Clearly, this pilot study is only the beginning of understanding the potential application of this serum tumour marker in canine mammary cancer management, and we hope that these findings will encourage future research work in this field.

## Competing interest

The authors declare that they have no competing interests.

## Authors’ contributions

EM and MK conceived the study and drafted the manuscript. EM performed the histopathology and the scoring process and supervised the immunohistochemical procedure. MK, KP and CC performed cell culture, immunocytochemistry and contributed to Western blot analysis. MK performed the 3D culture. KM performed the invasion assay. ADG and KF performed the Western blot. FF and MK performed the statistical analyses. MCM and EP performed the chemiluminescent assay. All authors read and approved the final manuscript.
